# Association Cystatin C and Risk of Stroke in Elderly Patients With Obstructive Sleep Apnea: A Prospective Cohort Study

**DOI:** 10.3389/fnins.2021.762552

**Published:** 2021-12-15

**Authors:** Xiaofeng Su, Yinghui Gao, Weihao Xu, JianHua Li, Kaibing Chen, Yan Gao, JingJing Guo, LiBo Zhao, Huanhuan Wang, Xiaoshun Qian, Junling Lin, Jiming Han, Lin Liu

**Affiliations:** ^1^Department of Pulmonary and Critical Care Medicine of the Second Medical Center and National Clinical Research Center for Geriatric Diseases, Chinese PLA General Hospital, Beijing, China; ^2^Medical College, Yan’an University, Yan’an, China; ^3^PKU-UPenn Sleep Center, Peking University International Hospital, Beijing, China; ^4^Cardiology Department of the Second Medical Center and National Clinical Research Center for Geriatric Diseases, Chinese PLA General Hospital, Beijing, China; ^5^Sleep Center, The Affiliated Hospital of Gansu University of Chinese Medicine, Lanzhou, China; ^6^Department of General Practice, 960th Hospital of PLA, Jinan, China; ^7^Sleep Medicine Center, Department of Respiratory and Critical Care Medicine, Peking University People’s Hospital, Beijing, China; ^8^Department of Pulmonary and Critical Care Medicine, Beijing Chaoyang Hospital Affiliated to Capital Medical University, Beijing, China

**Keywords:** Cystatin C, obstructive sleep apnea, elderly, stroke, cohort study

## Abstract

**Background:** Few prospective cohort studies have assessed the relationship between Cystatin C (Cys-C) and risk of stroke in elderly patients with obstructive sleep apnea (OSA). The study sought to examine the association between baseline serum Cys-C and long-term risk of stroke among elderly OSA patients.

**Methods:** A total of 932 patients with OSA, no history of stroke, ≥60 years of age, and complete serum Cys-C records were included in this study. All patients had completed polysomnography (PSG). OSA was defined as an apnea-hypopnea index (AHI) of ≥5 events per hour. Participants were categorized into four groups according to baseline serum Cys-C concentration, split into quartiles. Multivariate Cox regression were used to evaluate the association between Cys-C and the incidence of new-onset stroke.

**Results:** Stroke occurred in 61 patients during the median 42-month follow-up period. The cumulative incidence rate of stroke was 6.5%, which included 54 patients with ischemic stroke and 7 patients with hemorrhagic stroke. The cumulative incidence of stroke was higher among patients with baseline serum Cys-C concentration of ≥1.15 mg/L when compared with other groups (*P*_Log–rank_ < 0.001). After adjusting for potential confounding factors in the Cox regression model, patients with a serum Cys-C concentration of ≥1.15 mg/L had a 2.16-fold higher risk of developing stroke compared with patients with serum Cys-C ≤ 0.81 mg/L (HR, 2.16, 95%CI, 1.09–6.60; *P* = 0.017). Additionally, there was a higher risk in those of age ≥70 years (HR, 3.23, 95%CI, 1.05–9.24; *P* = 0.010). The receiver-operating characteristic curves showed that the capability of Cys-C to identify elderly patients with OSA who had a long-time risk of stroke was moderate (AUC = 0.731, 95% CI: 0.683–0.779, *P* = 0.001).

**Conclusion:** Increased Cys-C concentration was identified as a risk factor in the incidence of stroke in elderly patients with OSA, independent of gender, BMI, hypertension and other risk factors. Additionally, it conferred a higher risk in patients of age ≥70 years.

## Introduction

The number of patients with stroke is increasing annually. Local and international epidemiological surveys have focused on the high-risk factors of stroke. Accumulating evidence has revealed that obstructive sleep apnea (OSA) and Cystatin C (Cys-C) play important roles in the increased risk of stroke. Cys-C is an endogenous inhibitor of cysteine proteinases that is involved in the catabolism of intracellular proteins and peptides. It is an effective marker of renal function; its filtration rate from the blood is shorter than serum creatinine, which makes it capable of detecting declined renal function earlier (Yang et al., 2020; Wang et al., 2021). A recent meta-analysis has indicated a strong correlation between declined renal function and increased stroke risk; the presence of serum Cys-C may pose a substantial risk because the anatomical, vasoregulatory, and hemodynamic mechanisms of the brain and kidney are similar (Chelluboina and Vemuganti, 2019). Data from cross-sectional studies has confirmed that serum Cys-C is independently associated with increased risk of stroke (Guoxiang et al., 2018; Ren et al., 2020). However, prospective studies have produced conflicting results on the causal relationship between the incidence of stroke and baseline serum Cys-C concentration (van der Laan et al., 2016; Zhu et al., 2018; Guo et al., 2020).

As the most common form of sleep disordered breathing (SDB), OSA has been associated with the occurrence of stroke *via* multiple pathological mechanisms, such as vascular endothelial dysfunction, oxidative stress, inflammatory reaction, and promoting atherosclerosis (Mohammad et al., 2019). Structural changes in total gray matter volume have been linked with OSA; however, a decrease of gray matter volume related to OSA is rarely observed (Innes et al., 2015). In addition, the elderly are moderately tolerant to intermittent hypoxia and can resist a decrease in cerebral blood flow perfusion through ischemic preconditioning and neuroprotection in the early stages (Kaculini et al., 2020). Therefore, the incidence of stroke in elderly patients with mild to moderate OSA is not increased significantly (Catalan-Serra et al., 2019). Cys-C is a key determinant of endogenous neuroprotection in the brain. In pre-stroke lesions, the release of Cys-C is a neuroprotective response (Charlson et al., 1987; Kirchhof et al., 2016). There may be a common mechanism between OSA and Cys-C in promoting stroke; however, to the best of our knowledge, no study has assessed the relationship between serum Cys-C concentration and the long-term stroke risk in patients with OSA in a large prospective study. We hypothesize that stroke incidence in elderly patients with OSA may differ according to serum Cys-C concentration. Therefore, the high sensitivity of serum Cys-C may be a better predictor of the risk of stroke, which is currently hidden due intermittent hypoxia tolerance in this patient group.

Here, we performed a large-scale, multicenter, prospective cohort study and performed a survival analysis to delineate the association of baseline serum Cys-C concentration and the risk of stroke in elderly patients with OSA.

## Materials and Methods

### Patients and Design

**FIGURE 1:**
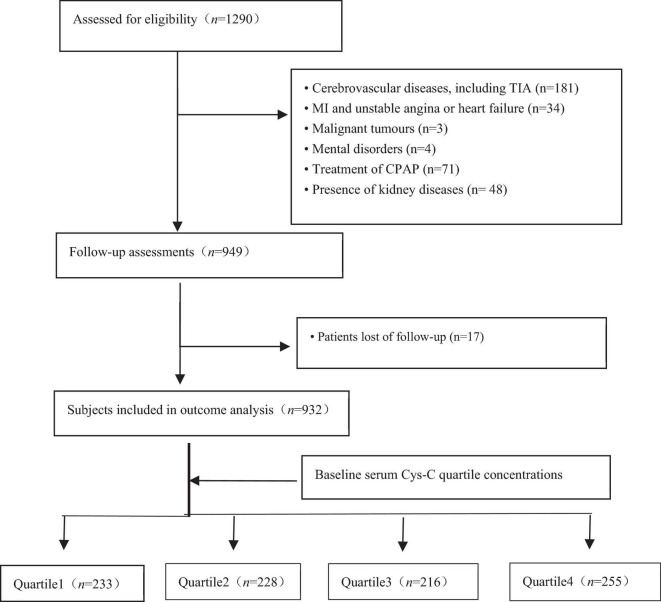
Study flowchart. MI: myocardial infarction; CPAP: continuous positive airway pressure.

From January 2015 to October 2017, a longitudinal cohort was formed that consisted of 1290 participants who received in an overnight polysomnography (PSG) examination at the Chinese PLA General Hospital, Peking University International Hospital, Peking University People’s Hospital, Beijing Chaoyang Hospital, 960th Hospital of PLA, or the affiliated Hospital of Gansu University of Chinese Medicine. The pre-specified outcome of interest was stroke. The study flowchart is presented in Figure 1. Consecutive patients with OSA, aged ≥60 years, no history of myocardial infarction (MI), hospitalization for unstable angina or heart failure were eligible for inclusion. OSA was defined as an apnea-hypopnea index of ≥5 events per hour through overnight PSG examinations. The AHI was defined as the number of apnea and hypopnea per hour of sleep. We excluded 358 patients based on the following criteria: (1) diagnosis of cerebrovascular diseases, including transient ischemic attack (TIA); (2) CPAP treatment for OSA; (3) previous history of myocardial infarction (MI), hospitalization for unstable angina or heart failure; (4) presence of malignant tumors; (5) presence of mental disorders; and (6) presence of kidney diseases. Furthermore, we excluded those lost during the follow-up; therefore, the final study population included 932 elderly patients with OSA. Our study was a large-scale, multicenter, prospective, cohort study that assessed the association between baseline serum Cys-C concentration and the incidence of stroke in elderly patients with OSA. Participants were divided into four groups based on baseline serum Cys-C quartile concentrations. This study conformed to the STROBE (Strengthening the Reporting of Observational studies in Epidemiology) guidelines and was carried out in accordance with the Declaration of Helsinki. The Ethics Committee of Chinese PLA General Hospital (S2020-397-02) approved the study. All participants provided written informed consent.

### Measurements of Covariates

All the study participants provided personal demographics, clinical characteristics, and sleep parameters. These data were assessed by an interviewer who administered the Unified epidemiological questionnaire and were reviewed by three physicians. Body mass index (BMI) was calculated as weight (kg) divided by height (m^2^). Smoking status was categorized as current smoker, former smoker (no smoking in the past 30 days), or never-smoker (smoking fewer than 100 cigarettes in the lifetime). Blood was drawn for biochemical analysis after overnight Sleep Study. At the baseline visit (within14 days after OSA), samples were frozen before being shipped on dry ice to be stored at a central lab at –80°C. Serum Cys-C were measured by latex immunoturbidimetric assays (Siemens, Germany, BNII SYSTEM) and conducted by laboratory personnel blinded to treatment allocation and clinical outcome at the PLA (Chinese PLA General Hospita) National Clinical Research Center for Geriatric Diseases Laboratory in Beijing, China. Systolic blood pressure (SBP) and diastolic blood pressure (DBP) were measured three times. The mean of the second and third measurements was used in the analysis. Hypertension was defined as SBP/DBP of ≥140/90 mmHg or the use of antihypertension medication. Diabetes was defined as a fasting glucose level of ≥126 mg/dL or the use of any hypoglycemic medication. Atrial fibrillation was defined based on the ESC 2016 guidelines (Fang et al., 2017). Carotid atherosclerosis, coronary heart disease (CHD) and chronic obstructive pulmonary disease (COPD) were determined by a record of a relevant diagnostic clinical (Read) code indicating the presence of the condition (Fang et al., 2019). The categories of covariates were listed in Supplementary Table 1.

### Overnight Polysomnography

All patients underwent an overnight sleep monitoring (from 21: 00 to 7: 00 the next day) after clinical stabilization during hospitalization at sleep center (within 1 weeks after admission) and sleep parameters were recorded using portable laboratory-based polysomnography (PSG) instrument (Compumedics, Melbourne, Australia), as described previously (Su et al., 2021). Recording channels were as follows: one electroencephalography (C4-A1), two electrooculography, two electrocardiography, one airflow from nasal airflow pressure transducer, two thoracic and abdominal impedance belts for respiratory effort, one pulse oximeter, and one position sensor, and one tracheal microphone for snoring (Kim et al., 2018). PSG records were automatically analyzed, manually calibrated by twice (by XFS and YHG), both of whom were blinded to the demographic and clinical characteristics, and further reviewed by a senior sleep physician. The apnea-hypopnea index (AHI) was defined as the number of apnea and hypopnea events per hour of sleep. The oxygen desaturation index (ODI) was defined as a SaO_2_ drop of ≥3%. OSA was classified as mild (AHI, 5–14.9), moderate (AHI, 15–30), or severe (AHI, >30) (Kapur et al., 2017).

### Follow-Up and Outcome

The study outcome was stroke, including ischemic stroke and hemorrhagic stroke. 932 patients with OSA were followed up from the diagnosed time of PSG assessment and patients or their proxies were contacted by telephone at 1 month, 3 months, 6 months, 1 year by two investigators who were blinded to patients’ PSG results every 6 months. The follow-up through the phone was to preliminary identify medical records for stroke or if reason was brain hemorrhage, brain aneurysm, trouble speaking, numbness, or other stroke symptoms, and then every 6 months thereafter (at least 3 months and up to 1 years) by a clinic visit and medical chart review to further diagnosis, which lasted until end of the study period (December 2020). CT or MRI Report was obtained using an outpatient physical examination for a clinic visit. Medical records were requested and examined for all individuals who had suspected strokes by at least 2 senior physicians of stroke experts to validate and classify potential strokes and were adjudicated by the clinical event committee.

After each follow-up, a multidisciplinary team of sleep medicine physicians, general practitioners, respiratory and critical care physicians, and cardiovascular physicians provided relevant health service suggestions based on the different symptoms or diseases of the patients. This was conducted by another group investigators who were blinded the outcome of the current follow-up. All patients received standard health care according to their disease status during follow-up. Patients with moderate and severe OSA were encouraged to use continuous positive airway pressure (CPAP) treatment.

The study ended if patients reported the incidence of a new-onset stroke, which was the first stroke event for that patient. Two or more strokes were uniformly counted as one stroke event, with the first stroke time and event reported as the outcome.

### Diagnosed of Stroke

Stroke was defined according to the Diagnostic Criteria of Cerebrovascular Diseases in China (2019) (Chinese Society of Neurology and Chinese Stroke Society, 2019). The prevalence of stoke was estimated as the percentage of participants who reported having had a stroke at baseline examination. The incidence of stroke was defined as the percentage of participants who were newly diagnosed with stroke during the median 42-month follow-up period excluding patients who had reported a stroke at baseline. Both ischemic and hemorrhagic stroke were included. The stroke were preliminary diagnosed by self-report of physician diagnosis by patients themselves and/or their proxies (spouse or child), further diagnosis is based on the CT or MRI report obtained from the outpatient visit and medical records evaluated by 2 physicians (including JHL and YG)Of a committee of stroke experts, and all stroke events were adjudicated by the clinical event committee.

### Statistical Analyses

Continuous variables are shown as mean ± SD or median (first and third quartiles) and were compared by using ANOVA or Kruskal-Wallis tests. Categorical variables are presented as counts (percentage) and were compared using Chi-square statistics or Fisher’s exact test. Cys-C was prospectively modeled as a continuous variable and reported categorically by quartile. Kaplan-Meier curves were used to visualize the association between serum Cys-C concentration quartiles and stroke events. Cox proportional hazard regression analysis was performed to assess the hazard ratios for stroke according to the serum Cys-C concentration quartiles in elderly patients with OSA. Model 1 was unadjusted. In model 2, analyses were partially adjusted for age, sex, and body mass index (BMI). Model 3 was fully adjusted for potential confounders, including age, gender, BMI, SBP, DBP, smoking, drinking, triglycerides (TG), uric acid, total cholesterol (TC), total sleep time (TST), coronary heart disease (CHD), chronic obstructive pulmonary disease (COPD), glucose, and hypertension. Adjusted hazard ratios were estimated with 95% confidence intervals (CI). Patients who reported a stroke within 12 months of follow-up were excluded and re-analyzed in Model 4 to rule out reverse causality. Sensitivity analyses modeled Cys-C as a dichotomous variable [fourth versus first 3 quartiles (Q4: Q1–3)] or three classification variable [fourth versus third versus first 2 quartiles (Q4: Q3: Q1–2)]. The adjusting confounder factors of Model 4 were the same as Model 3. The receiver-operating characteristic curve was plotted to estimate the capability of serum Cys-C to discriminate patients with a risk of stroke. Statistical significance was identified as *P* < 0.05. All analyses were conducted using the SPSS (version 25.0, SPSS Inc., Chicago, IL, United States).

## Results

### Baseline Characteristics

In total, 1290 consecutive eligible patients with OSA aged ≥60 years were prospectively enrolled, all of whom underwent a successful overnight sleep study. Of the 1290 participants, follow-up was available on 1273 (98.7%). After exclusion of patients according to predefined criteria, 932 study subjects were included in the final analysis (Figure 1); descriptive characteristics of the patients with OSA by quartile of serum Cys-C concentration are shown in Table 1. The median age was 66 (range, 60–96) years, and 60.6% of patients were male. Gender, BMI, SBP, TG, smoking, creatinine, uric acid, HDL, left ventricular ejection fraction (LVEF), TST, hyperlipidemia, hypertension, diabetes, atrial fibrillation, and carotid atherosclerosis were significantly different between groups (*P* < 0.05). There were no differences in age, DBP, drinking, glucose, TC, LDL, direct bilirubin, waist circumference, neck circumference, waist-hip ratio, COPD, CHD, and other sleep parameters between groups.

**TABLE 1:** Characteristics of elderly OSA participants by Cys-C stratum.

Variables	Quartile 1 (*n* = 233)	Quartile 2 (*n* = 228)	Quartile 3 (*n* = 216)	Quartile 4 (*n* = 255)	*P*-Value
Age [year, *M(Q_1_, Q_3_)*]	65 (62, 71)	67 (64, 73)	66 (64, 72.5)	67 (63, 73)	0.06
male [*n (%)*]	105 (47.1)	128 (59.5)	143 (62.7)	189 (71.1)	<0.001
BMI [kg/m^2^, *M(Q_1_, Q_3_*)]	25.51 (22.86, 28.41)	24.4 (22.49, 27.7)	25.63 (22.95, 28.56)	26.67 (24.29, 31.69)	0.021
SBP [mmHg, *M(Q_1_, Q_3_*)]	136 (120, 150)	138 (123, 150)	136 (122, 152)	138 (125,150)	0.024
DBP [mmHg, *M(Q_1_, Q_3_)*]	80 (69, 88)	77 (70, 90)	76 (70, 85)	80 (70, 85)	0.221
Current smoker[*n (%)*]	43 (19.3)	40 (18.6)	41 (18.0)	67 (25.2)	0.023
Current drinker [*n (%)*]	17 (7.6)	15 (7.0)	29 (12.7)	36 (13.5)	0.129
Creatinine [μmol/L, *M(Q_1_, Q_3_)*]	61 (53, 73)	67 (59, 74)	69 (60, 80)	79 (68, 99)	<0.001
Uric acid [μmol/L, *M(Q_1_, Q_3_*)]	264 (227, 318)	296 (248,336)	325 (262, 358)	391 (344, 447)	<0.001
Glucose [mmol/L, *M(Q_1_, Q_3_)*]	5.70 (4.97, 6.93)	5.60 (4.95, 6.90)	5.69 (5.20, 6.66)	5.55 (5.04, 6.60)	0.088
TC [mmol/L, *M(Q_1_, Q_3_)*]	4.43 (3.68, 5.25)	4.20 (3.64, 5.15)	4.14 (3.44, 4.71)	4.15 (3.41, 4.87)	0.078
TG [mmol/L, *M(Q_1_, Q_3_)*]	1.24 (0.94, 1.74)	1.26 (0.93, 1.78)	1.25 (0.89, 1.80)	1.46 (1.01, 2.02)	0.013
HDL [mmol/L, *M(Q_1_, Q_3_)*]	1.17 (0.98, 1.48)	1.14 (1.01, 1.45)	1.16 (0.94, 1.30)	1.06 (0.91, 1.31)	0.000
WC[cm, *M(Q_1_, Q_3_)*]	94.0 (80.5, 102.5)	89.0 (80.0, 98.5)	91.0 (78.0, 100.5)	99.0 (90.0, 113.0)	0.596
NC [cm, *M(Q_1_, Q_3_)*]	38.0 (34.5, 41.0)	38.0 (35.0, 40.0)	37.50 (35.0, 43.0)	38.0 (36.0, 41.50)	0.980
WHR [%, *M(Q_1_, Q_3_)*]	0.9 (0.8, 1.1)	0.9 (0.8, 1.3)	0.9 (0.8, 1.1)	0.9 (0.8, 1.4)	0.587
LDL [mmol/L, *M(Q_1_, Q_3_)*]	2.19 (1.90, 3.03)	2.30 (1.78, 2.86)	2.45 (1.93, 3.09)	2.37 (1.81, 2.810	0.098
DBil [μmol/L, *M(Q_1_, Q_3_)*]	3.85 (2.98, 4.70)	3.60 (2.60,5.30)	3.85 (3.10, 5.30)	3.60 (2.91, 4.88)	0.883
LVEF [%, *M(Q_1_, Q_3_)*]	64.0 (60.0, 69.3)	64.0 (60.5, 71.2)	65.0 (66.4, 68.5)	64.5 (60.9, 67.0)	0.024
TST [h, *M(Q_1_, Q_3_)*]	6.60 (5.77, 7.30)	6.80 (6.17, 7.40)	7.00 (6.43, 7.50)	7.00 (6.12, 7.60)	0.008
AHI [times/h, *M(Q_1_, Q_3_)*]	28.60 (14.30, 43.90)	29.30 (12.80, 46.90)	26.90 (15.21, 47.75)	28.70 (15.62, 45.63)	0.957
ODI [times/h, *M(Q_1_, Q_3_*)]	16.80 (9.40, 36.00)	21.01 (9.11, 42.10)	19.02 (8.55, 39.83)	21.70 (10.01, 39.80)	0.907
MSpO2 [*%*, *M(Q_1_, Q_3_)*]	94 (92, 95)	94 (91, 95)	94 (92, 95)	94 (92, 95)	0.668
LSpO2 [*%, M(Q_1_, Q_3_)*]	81 (75, 86)	81 (73, 85)	81 (73, 86)	80 (70, 85)	0.512
TSA90 [min, *M(Q_1_, Q_3_)*]	8.51 (0.81, 46.42)	7.03 (0.55, 54.23)	10.71 (1.22, 46.16)	8.3 (2.21,30.79)	0.970
Hyperlipidemia [*n (%)*]	41 (25.5)	63(29.3)	78 (34.2)	98 (31.8)	0.007
Hypertension [*n (%)*]	131 (58.7)	140 (65.1)	148 (64.9)	191 (71.8)	0.026
Atrial fibrillation [*n (%)*]	6 (2.7)	15 (7.0)	15 (6.6)	30 (11.3)	0.003
Carotid atherosclerosis [*n (%)*]	67 (30)	50 (23.3)	67 (29.4)	99 (37.2)	0.011
COPD [*n (%)*]	19 (8.5)	10 (4.7)	19 (8.3)	29 (10.9)	0.104
Diabetes [*n (%)]*	46 (20.6)	53 (24.7)	63 (27.6)	88 (33.1)	0.016
CHD [*n (%)*]	39 (17.5)	52 (24.2)	53 (23.2)	75 (28.2)	0.052

*BMI: body mass index; SBP: systolic blood pressure; DBP: diastolic blood pressure; HDL: high-density lipoprotein; LDL: low-density lipoprotein; DBil: direct bilirubin; LVEF: left ventricular ejection fraction; WC: waist circumference; NC: neck circumference; WHR: waist-hip ratio; AHI: the apnea-hypopnea index; ODI: the oxygen desaturation index; MSpO2: the mean pulse oxygen saturation; LSpO2: the lowest pulse oxygen saturation; TSA90: the duration of time with SaO2 < 90%; TST: total sleep time; TG: triglyceride; TC: total cholesterol; OSA: obstructive sleep apnea; CHD: coronary heart disease; COPD: chronic obstructive pulmonary disease; Quartile 1: Cys-C ≤ 0.81 mg/L; Quartile 2: 0.8 < Cys-C < 0.97 mg/L; Quartile 3: 0.97 ≤ Cys-C < 1.15 mg/L; Quartile 4: Cys-C ≥ 1.15 mg/L.*

### Association Between the Risk of Stroke and Cystatin-C in Patients With Obstructive Sleep Apnea

**FIGURE 2:**
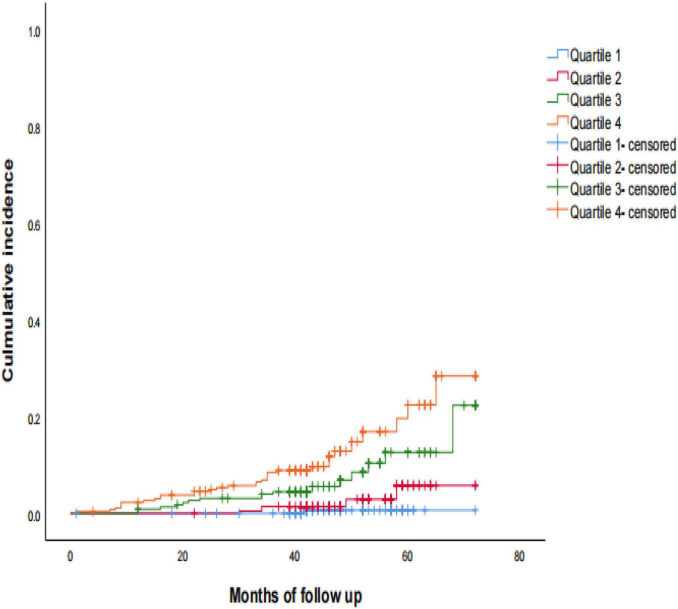
Kaplan-Meier estimates of probability of cumulative incidence (%) for stroke. Log-rank test: *P* < 0.001; Quartile 1 was used as reference group.

During a median follow-up of 42 months (range, 1–72 months), 61 (6.5%) patients had a stroke: 54 and 7 reported having an ischemic (5.8%) and hemorrhagic (0.8%) stroke, respectively. Kaplan-Meier analysis of the cumulative survival rate of stroke according to baseline serum Cys-C concentration quartiles at the 42-month follow-up period are shown in Figure 2. The cumulative survival of stroke was the lowest among patients in Quartile 4 (*P*_Log–rank_ < 0.001). Interestingly, the incidence of stroke increased in a dose-dependent manner according to the baseline concentration of serum Cys-C: Quartile 1 (0.4%) vs. Quartile 2 (4.3%) vs. Quartile 3 (7.9%) vs. Quartile 4 (13.3%; *P* < 0.001).

**FIGURE 3:**
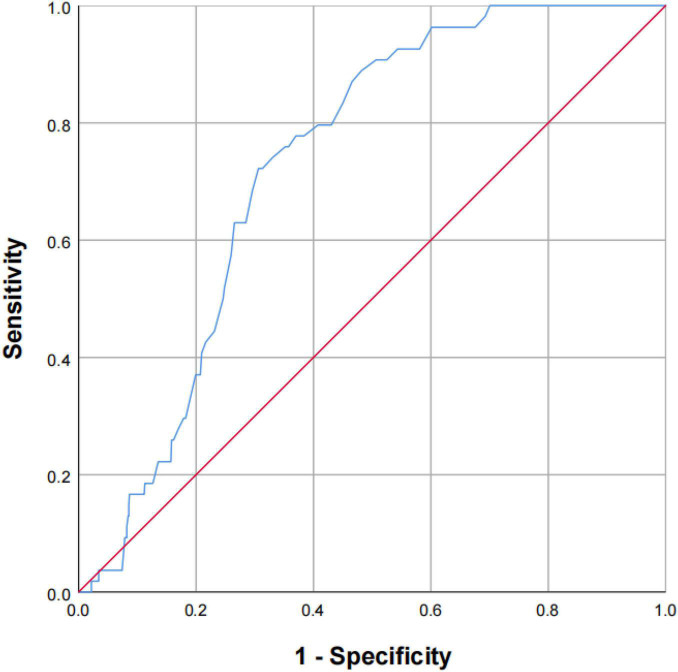
The capability of Cys-C to identify the patients of OSA with a high risk of stroke. Receiver-operating characteristic curve showed the performance of Cys-C in predicting a high risk of stroke. The AUC was 0.731 (95% CI: 0.683–0.779, *P* = 0.001). AUC: area under the receiver operating characteristic curve; Cys-C: cystatin C; CI: confidence interval.

Next, multivariate analysis was performed to better assess the risk of stroke events. After multivariable adjustment, the strength of the relationships was attenuated, but increasing concentration of Cys-C remained significantly associated with a 16% higher hazard of stroke, which Quartile 4 had a 2.16-fold higher risk of developing stroke compared with those Quartile 1 (adjusted HR, 2.16; 95% CI, 1.09–6.60; *P* = 0.017; Table 2). Baseline serum Cys-C concentration was moderately capable at identifying patients with a long-term risk of stroke (AUC = 0.731; 95% CI: 0.683–0.779, *P* = 0.001; Figure 3). Furthermore, analysis revealed a stronger association between Cys-C and risk of stroke in patients ≥70 years of age (HR, 3.23; 95%CI, 1.05–9.24; *P* = 0.010), after adjustment for age, sex, and other confounding factors (Table 3).

**TABLE 2:** Association between Cys-C and incidence of stroke.

Group	Model 1		Model 2		Model 3	
	HR (95%CI)	*P*-Value	HR (95%CI)	*P*-Value	HR (95%CI)	*P*-Value
Quartile 1	1.00 (ref.)		1.00 (ref.)		1.00 (ref.)	
Quartile 2	1.05 (0.75, 1.75)	0.347	1.14 (0.16, 1.86)	0.326	1.59 (0.59, 4.31)	0.352
Quartile 3	2.11 (1.04, 2.98)	0.036	2.33 (1.38, 2.78)	0.041	2.17 (0.85, 5.52)	0.104
Quartile 4	2.52 (1.57, 5.95)	0.018	2.49 (1.45, 5.96)	0.023	2.16 (1.09, 6.60)	0.017

*Hazard ratios (HRs) with 95% confidence intervals from Cox regression analysis.*

*Model 1: unadjusted for the Cys-C group.*

*Model 2: adjusted for the Cys-C group, age, gender, and BMI.*

*Model 3: adjusted for the Cys-C group, age, gender, BMI, SBP, DBP, smoking, drinking, TST, TC, creatinine, HDL, LDL, DBil, LVEF, uric acid, TG, CHD, COPD, glucose, hypertension, atrial fibrillation, carotid atherosclerosis, hyperlipidemia, and diabetes.*

**TABLE 3:** Subgroup analysis of the associations between Cys-C and incidence of stroke.

		Quartile 1		Quartile 2		Quartile 3		Quartile 4	
		HR (95%CI)	*P*-Value	HR (95%CI)	*P*-Value	HR (95%CI)	*P*-Value	HR (95%CI)	*P*-Value
**Aged ≥70**									
	Model 1	1.00 (ref.)		2.44 (1.06, 5.76)	0.036	2.34 (1.04, 3.69)	0.048	2.24 (0.91, 3.51)	0.044
	Model 2	1.00 (ref.)		2.70 (1.36, 6.51)	0.025	2.68 (1.23, 3.68)	0.031	2.31 (1.15, 3.48)	0.039
	Model 3	1.00 (ref.)		3.83 (1.38, 9.93)	0.009	3.76 (1.23, 9.92)	0.008	3.23 (1.05, 9.24)	0.010
**Age <70**									
	Model 1	1.00 (ref.)		1.09 (0.39, 2.22)	0.353	1.03 (0.38, 2.29)	0.214	1.01 (0.33, 2.17)	0.305
	Model 2	1.00 (ref.)		1.12 (0.44, 2.87)	0.136	1.24 (0.41, 3.12)	0.122	1.15 (0.37, 2.89)	0.131
	Model 3	1.00 (ref.)		2.13 (0.84, 5.01)	0.138	2.22 (0.88, 5.31)	0.126	1.93 (0.69, 4.52)	0.210

*Hazard ratios (HRs) with 95% confidence intervals from Cox regression analysis.*

*Model 1: unadjusted for the Cys-C group.*

*Model 2: adjusted for the Cys-C group, age, gender, and BMI.*

*Model 3:adjusted for the Cys-C group, age, gender, BMI, SBP, DBP, smoking, drinking, TST, TC, creatinine, HDL, LDL, DBil, LVEF, uric acid, TG, CHD, COPD, glucose, hypertension, atrial fibrillation, carotid atherosclerosis, hyperlipidemia, and diabetes.*

### Sensitivity Analysis

To rule out the possibility of reverse causality, elderly patients who reported having a stroke within 12 months of follow-up were excluded and re-analyzed in Model 4. The adjusted confounding factors of Model 4 were the same as Model 3. The multivariate Cox regression analysis showed that patients in Quartile 4 had a 2.01-fold higher high risk of stroke than those in Quartile 1, after adjusting for age, gender, BMI, SBP, DBP, smoking, drinking, TST, TC, creatinine, HDL, LDL, DBil, LVEF, uric acid, CHD, COPD, glucose, hypertension, atrial fibrillation, carotid atherosclerosis, hyperlipidemia, and diabetes risk factors (adjusted HR, 2.01; 95% CI, 1.21–6.97; *P* = 0.029). Furthermore, we also made a comparative analysis of 2 or 3 groups, and when Cys-C were dichotomized (Cys-C Q4:Q1–Q3) and entered simultaneously in a model adjusting for other covariates, higher concentrations of Cys-C remained associated with a 92% higher hazard of stroke (adjusted HR Q4:Q1–Q3 1.92, 95% CI 1.14–3.23, *P* = 0.015). Similarly, Cys-C were divided into 3 groups (Cys-C Q4:Q3:Q1–Q2) also remained associated with a 72% higher hazard of stroke increasing concentration of Cys-C compared to control group (Q1–Q2) (adjusted HR Q4:Q1–Q3 2.72, 95% CI 1.41–5.26, *P* = 0.003), Supplementary Table 2.

## Discussion

To the best of our knowledge, this is the first study to show that the baseline serum Cys-C concentration is correlated with the incidence of stroke in a large population of elderly patients with OSA. Cys-C is a cost-effective clinical biochemical marker that provides an objective measurement that is not affected by inflammation, liver disease, diet, and individual constitution. It is widely used, easy to obtain, and a more accurate marker of renal function than serum creatinine (Coll et al., 2000; Martinez-Vargas et al., 2014). A previous study has shown that Cys-C is an independent risk factor for post-stroke death and poor prognosis in patients with chronic kidney disease (CKD) (Yahalom et al., 2009). Additionally, Cys-C is an independent risk factor of cerebrovascular disease among the elderly without kidney disease (Guoxiang et al., 2018; Huang et al., 2019). However, the causal relationship between OSA and baseline serum Cys-C concentration in relation to long-term stroke risk remains unclear.

In this study, we found a significant correlation between baseline serum Cys-C and the risk of long-term stroke in elderly patients with OSA: participants with higher baseline serum Cys-C concentrations had a lower cumulative survival rate of stroke. Further, those in Quartile 4 (≥1.15 mg/L) had over three times higher risk of stroke than those in Quartile 1, after adjusting for confounders. Furthermore, we observed that Cys-C was moderately capable of identifying patients with a long-term risk of stroke, independent of age, gender, BMI, and other risk factors.

Cystatin-C has an endogenous neuroprotective effect in the brain, which could mediate the exogenous non-ischemic preconditioning mechanism to protect against cerebrovascular injury (Kirchhof et al., 2016). In the early stages of stroke, the body’s self-compensation effect leads to an increase in Cys-C release (Martinez-Vargas et al., 2014). We excluded the patients who reported stroke events within 12 months of follow-up to minimize the influence of reverse causality for our study. These were then re-analyzed independently, which showed that those in Quartile 4 had a threefold higher risk for developing stroke compared with those in Quartile 1. This further confirmed the reliability of our study and our findings.

Cystatin-C is a protein encoded by the CST3 gene. It is a cysteine protease inhibitor produced by all nucleated cells at a constant rate; therefore, it exists in all tissues and body fluids (Sawada, 2021). It is especially abundant in the central nervous system, with fivefold higher concentrations than in the blood (Kirchhof et al., 2016). Our study found no significant difference in the long-term risk of stroke in elderly patients with OSA who had Cys-C concentrations in Quartiles 2 and 3 compared with Quartile 1; however, but the cumulative survival rate showed a decreasing trend.

A Cys-C concentration of ≥1.15 mg/L increased the risk of stroke. This may be due to several possible pathological mechanisms. First, Cys-C plays an important role in atherosclerosis, which can inhibit the hydrolysis activity of cathepsin-dependent proteins in blood vessel wall. The remodeling of the vascular wall extracellular matrix (ECM) is an important feature in the pathogenesis of atherosclerosis. An imbalance between the production of cathepsin can increase neuronal apoptosis in the brain, which increases Cys-C release. This reduces neuronal injury but leads to an increase in ECM degradation, which aggravates the occurrence and development of atherosclerotic plaques and increases the risk of stroke (Martinez-Vargas et al., 2014; Nishimura et al., 2021). Second, the soft tissue vibration in the upper respiratory tract, caused by snoring and repeated intermittent hypoxia trigger the participation of hypoxia-inducible factor in the formation of atherosclerotic plaque (Kirkham et al., 2017; Deeb et al., 2019). Third, OSA is considered to induce a persistent, low-intensity, inflammatory state. One of inflammatory pathway, CD40-CD40L, enhances leukocyte recruitment to the sites of vascular inflammation and stimulates macrophages to produce matrix metalloproteinase (MMP). This process is associated with atherosclerotic plaque formation (Li et al., 2020; Migacz et al., 2021). Fourth, Cys-C is also known as neutrophil growth factor and is involved in the migration, phagocytosis, and inflammation of neutrophils. It directly stimulates the synthesis of vascular smooth muscle cells and release of cathepsin, which can cause the elastic tissue in the damaged artery to separate and aggravates vascular wall injury. This leads to an increase in atherosclerotic plaque load and plaque formation (Li et al., 2020; Ren et al., 2020). Further, this can indirectly worsen the course of OSA and increase the risk of stroke *via* inflammatory pathway mechanisms.

The clinical positive symptoms of elderly patients of OSA, such as the daytime sleepiness and obesity, can be concealed. Furthermore, the sense of suffocation at night and subjective sleep problems are not prominent. One study has shown that airway collapse in elderly patients with OSA is more likely than in young patients with OSA; however, the degree of dyspnea in clinical manifestations is lower (Edwards et al., 2014; Borker et al., 2021). Taken together with our data, which demonstrated a higher association between Cys-C concentration and risk of stroke in patients aged ≥70 years, this evidence should provide a feasible clinical basis for the “early detection” of stroke in elderly patients with OSA.

## Limitations

The strengths of this study included its multicenter, prospective design; large study population; the use of the gold standard method to assess stroke; PSG assessment of OSA; and the measurement of many potential confounding factors that could be accounted for in the fully adjusted models. However, several limitations should be acknowledged. First, some patients did not report new-onset stroke events during a median follow-up of 42 months; therefore, the follow-up time should be extended to verify the study conclusion. Second, the incidence of stroke is a complex process and is correlated with multiple factors. Although we adjusted for as many variables related to stroke as possible, there may still be other relevant factors we did not consider, such as hyperlipidemia (Menet et al., 2018). Third, our study was a multi-center and recruited primarily East-Asian patients, which may lead to unexpected selection bias. Fourth, there is no subgroup analysis of different stroke type because of too few cases. Accordingly, future longitudinal studies are required to address this issue. We do not believe that these limitations negate the value of our study.

## Conclusion

Our study shows that higher Cys-C that a sensitive biochemical marker of renal function exhibited a association in elderly patients of OSA and the development risk of stroke and to a higher extent in those greater than or equal to 70 years of age. Cys-C had a moderate ability to identify elderly patients of OSA with a high risk of stroke, independent of age, gender, BMI, and other risk factors. Therefore, preventive therapeutic strategies in elderly OSA individuals with a high baseline level of Cys-C may be effective to slow down the development risk of stroke in the future.

## Data Availability Statement

The raw data supporting the conclusions of this article will be made available by the authors, without undue reservation.

## Ethics Statement

The studies involving human participants were reviewed and approved by The Ethics Committee of Chinese PLA General Hospital (S2020-397-02). The patients/participants provided their written informed consent to participate in this study.

## Author Contributions

XS, YhG, WX, JHL, KC, YG, JG, LZ, and HW collected the data. XS and YhG analyzed the data and wrote the manuscript draft. JlL, JH, and LL designed this study. All authors have read and approved the manuscript.

## Conflict of Interest

The authors declare that the research was conducted in the absence of any commercial or financial relationships that could be construed as a potential conflict of interest.

## Publisher’s Note

All claims expressed in this article are solely those of the authors and do not necessarily represent those of their affiliated organizations, or those of the publisher, the editors and the reviewers. Any product that may be evaluated in this article, or claim that may be made by its manufacturer, is not guaranteed or endorsed by the publisher.

## Abbreviations

OSA: Obstructive sleep apnea
Cys-C: Cystatin C
PSG: Polysomnography
AHI: The apnea-hypopnea index
BMI: Body mass index
SBP: Systolic blood pressure
DBP: Diastolic blood pressure
ODI: The oxygen desaturation index
MSpO2: The mean pulse oxygen saturation
LSpO2: The lowest pulse oxygen saturation
CHD: Coronary heart disease
COPD: Chronic obstructive pulmonary disease
MI: Myocardial infarction
CPAP: Continuous positive airway pressure
ECM: Extracellular matrix
MMP: Matrix metalloproteinase
CKD: Chronic kidney disease.
