# Supplementary material for: Association Cystatin C and Risk of Stroke in Elderly Patients With Obstructive Sleep Apnea: A Prospective Cohort Study
Source: Front Neurosci. 2021 Dec 15;15:762552. doi: 10.3389/fnins.2021.762552 (PMC8715090; doi:10.3389/fnins.2021.762552)
Supplement: Supplementary file 1 [file Data_Sheet_1.docx]

Supplementary Table S-1 Characteristics of covariates

| Covariates | Category | Definition |
| --- | --- | --- |
| Sex, n (%) | Categorical variables | Male, female |
| Age, year | Continuous variable |  |
| BMI, kg/m^2^ | Continuous variable |  |
| SBP, mmHg | Continuous variable |  |
| DBP, mmHg | Continuous variable |  |
| Current smoker, n (%) | Categorical variables | Never smoking, Ever or Current smoking |
| Current drinker , n (%) | Categorical variables | Never alcohol drinking, Ever or Current alcohol drinking |
| Glucose, mmol/L | Continuous variable |  |
| Creatinine, μmol/L | Continuous variable |  |
| Uric acid , μmol/L | Continuous variable |  |
| Waist circumference, cm | Continuous variable |  |
| neck circumference, cm | Continuous variable |  |
| Waist-hip ratio | Continuous variable |  |
| HDL, mmol/L | Continuous variable |  |
| LDL, mmol/L | Continuous variable |  |
| DBil, μmol/ | Continuous variable |  |
| LVEF, % | Continuous variable |  |
| TST, h | Continuous variable |  |
| AHI, events/h | Continuous variable |  |
| ODI, events/h | Continuous variable |  |
| MSpO_2_, % | Continuous variables |  |
| LSpO_2_, % | Continuous variables |  |
| TSA90, min | Continuous variables |  |
| CHD, n (%) | Categorical variables | Yes, No |
| Hyperlipidemia, n (%) | Categorical variables | Yes, No |
| Hypertension, n (%) | Categorical variables | Yes, No |
| Atrial fibrillation, n (%) | Categorical variables | Yes, No |
| Carotid atherosclerosis, n (%) | Categorical variables | Yes, No |
| COPD, n (%) | Categorical variables | Yes, No |
| Diabetes | Categorical variables | Yes, No |

Table S-2 Association between different Cys-C groups and incidence of  stroke

|  | Unadjusted analysis | | Adjusted analysis | |
| --- | --- | --- | --- | --- |
|  | *HR* (95*%CI* ) | *P*-Value | *HR* (95*%CI* ) | *P*-Value |
| Two groups |  |  |  |  |
| Group A(Q1‐3) | 1.00 (ref.) | | 1.00 (ref.) | |
| Group B(Q4) | 2.21 (1.32, 3.68) | 0.002 | 1.92 (1.14, 3.23) | 0.015 |
| Three groups |  |  |  |  |
| Group C(Q1‐2) | 1.00 (ref.) | | 1.00 (ref.) | |
| Group D (Q3） | 2.25 (1.11, 4.59) | 0.025 | 2.18 (1.07, 4.45) | 0.031 |
| Group E (Q4) | 3.21 (1.68, 6.13) | 0.013 | 2.72(1.41, 5.26) | 0.003 |
